# Quorum Sensing Peptides Selectively Penetrate the Blood-Brain Barrier

**DOI:** 10.1371/journal.pone.0142071

**Published:** 2015-11-04

**Authors:** Evelien Wynendaele, Frederick Verbeke, Sofie Stalmans, Bert Gevaert, Yorick Janssens, Christophe Van De Wiele, Kathelijne Peremans, Christian Burvenich, Bart De Spiegeleer

**Affiliations:** 1 Drug Quality and Registration (DruQuaR) group, Faculty of Pharmaceutical Sciences, Ghent University, Ghent, Belgium; 2 Department of Radiology and Nuclear Medicine, Faculty of Medicine and Health Sciences, Ghent University Hospital, Ghent, Belgium; 3 Department of Medical Imaging, Medicine and Clinical Biology of Small Animals, Faculty of Veterinary Medicine, Ghent University, Merelbeke, Belgium; 4 Comparative Physiology and Biometrics, Faculty of Veterinary Medicine, Ghent University, Merelbeke, Belgium; Texas A&M University Health Science Center College of Medicine & Baylor Scott and White Health, UNITED STATES

## Abstract

Bacteria communicate with each other by the use of signaling molecules, a process called ‘quorum sensing’. One group of quorum sensing molecules includes the oligopeptides, which are mainly produced by Gram-positive bacteria. Recently, these quorum sensing peptides were found to biologically influence mammalian cells, promoting *i*.*a*. metastasis of cancer cells. Moreover, it was found that bacteria can influence different central nervous system related disorders as well, *e*.*g*. anxiety, depression and autism. Research currently focuses on the role of bacterial metabolites in this bacteria-brain interaction, with the role of the quorum sensing peptides not yet known. Here, three chemically diverse quorum sensing peptides were investigated for their brain influx (multiple time regression technique) and efflux properties in an *in vivo* mouse model (ICR-CD-1) to determine blood-brain transfer properties: PhrCACET1 demonstrated comparatively a very high initial influx into the mouse brain (K_in_ = 20.87 μl/(g×min)), while brain penetrabilities of BIP-2 and PhrANTH2 were found to be low (K_in_ = 2.68 μl/(g×min)) and very low (K_in_ = 0.18 μl/(g×min)), respectively. All three quorum sensing peptides were metabolically stable in plasma (*in vitro*) during the experimental time frame and no significant brain efflux was observed. Initial tissue distribution data showed remarkably high liver accumulation of BIP-2 as well. Our results thus support the potential role of some quorum sensing peptides in different neurological disorders, thereby enlarging our knowledge about the microbiome-brain axis.

## Introduction

Bacteria communicate by means of chemicals (*i*.*e*. pheromones), produced and released by the bacteria and recognized by others. Once a threshold concentration of these molecules is reached, a coordinated change in bacterial behavior is initiated. This process of cell-to-cell communication is called ‘quorum sensing’. Gram-negative and Gram-positive bacteria have different quorum sensing systems, each activated by specific quorum sensing molecules: N-acyl homoserine lactones (AHLs) trigger LuxI/LuxR circuits in Gram-negative bacteria, while Gram-positive bacteria mostly use peptides as signal molecules; both Gram-negative and Gram-positive bacteria produce autoinducer-2 (AI-2) family molecules [1,2]. Although not yet investigated, the presence of quorum sensing peptides in the human body is very likely, as shown by the biological incidence of Gram-positive bacteria and the *in vivo* detection of biofilms and AHL molecules in human feces, sputum and saliva [3–5]. The quorum sensing signaling molecules were originally found as intra-species communication tools in bacteria, but recent evidence indicates interspecies and host signaling as well [6–8]. The *Bacillus subtilis* quorum sensing peptide CSF (Competence and Sporulation Factor) activates p38 mitogen-activated protein kinase and protein kinase B (Akt) in host intestinal epithelial cells and induces cytoprotective heat shock protein synthesis [9]. Moreover, investigations from our group have indicated a selective crosstalk phenomenon between these quorum sensing peptides and mammalian cells: some quorum sensing peptides enhance breast or colon cancer cell invasion and promote angiogenesis, thereby potentially influencing cancer metastasis [10,11].

The brain is protected by a physiological barrier between the bloodstream and the central nervous system, *i*.*e*. the blood-brain barrier (BBB). This barrier is formed by the endothelium lining of the brain capillaries, possessing intercellular tight junctions, pericytes within the capillary basement membrane and astrocyte endfeet [12,13]. Research towards peptide-based therapeutics has been motivated by the discovery of the role of different neuropeptides in several neurological disorders. However, due to the presence of the BBB, inadequate delivery of these medicinally promising peptides to the brain is frequently observed. Currently, a number of peptides are used or investigated for their therapeutic purposes in *e*.*g*. epilepsy, pain, depression or brain cancer [14,15]. Increased success of peptide-based therapies is held to be due to the disruption of the BBB, which occurs in many neurological disorders, including brain cancer. However, in the early stages of brain neoplasms, the BBB is generally intact, so early treatment is limited, though indispensable [16,17].

Some of the neurological diseases are associated with an altered microbiota composition. In rodent models of anxiety, depression and autism like behavior a disrupted microbiome was observed [18–20]. Stress hormones (corticosterone and adrenocorticotropic hormone) also rose in germ-free mice compared to normal control mice when exposed to the same stress conditions and pretreatment with *Bifidobacterium infantis* induced more normal hormonal responses, again indicating the influence of the microbiome on stress responses [21,22]. Although current research mainly focuses on the role of neuroactive compounds such as neurotransmitters and metabolites, produced by the bacteria and acting on the brain [23–25]; the possible role of the quorum sensing peptides on these diseases deserves exploration, judging from the known exceptional activities of other peptides on the central nervous system.

Based on our previous results of bacterial quorum sensing peptide interactions with mammalian cells, together with the possible link between some neurological disorders and the host microbiome, we determined the blood-brain transfer of three chemically diverse quorum sensing peptides: if sufficient BBB-transport is observed, quorum sensing peptides can potentially contribute to the development of several brain pathologies.

## Materials and Methods

### Reagents

Calcium dichloride dihydrate, magnesium sulphate, potassium chloride, sodium chloride, sodium dihydrogen phosphate hydrate, sodium lactate and urethane were purchased from Sigma-Aldrich (Diegem, Belgium), while Bovine Serum Albumin (BSA), disodium hydrogen phosphate dihydrate, sodium iodide, sodium dihydrogen phosphate monohydrate, sodium metabisulphite and Chloramine-T were obtained from Merck KGaA (Darmstadt, Germany). Calcium dichloride, D-glucose, formic acid (FA) and HEPES were purchased from Fluka (Diegem, Belgium) and dextran from AppliChem GmbH (Darmstadt, Germany). For the mobile phases, acetonitrile was obtained from Fisher Scientific (Erembodegem, Belgium) and water was purified using an Arium 611 Pro VF purification system (Sartorius, Göttingen, Germany) to laboratory-graded water (18.2 MΩ × cm). For the radiolabeling of the peptides, Iodo-Gen^®^ coated tubes were purchased from Thermo Scientific (Erembodegem, Belgium) and the radioactive sodium iodide solution (Na^125^I) from Perkin Elmer (Zaventem, Belgium).

### Ethics Statement

Female, Institute for Cancer Research, Caesarean Derived-1 (ICR-CD-1) mice (Harlan Laboratories, Venray, The Netherlands) of age 7–10 weeks and weighing 25–34 g, were used during the BBB-transport experiments. All animal experiments were performed in strict accordance with the Belgian legislation RD 31/12/2012 and the Ethical Committee principles of laboratory animal welfare; the protocol was approved by the Ethical Committee of Ghent University, Faculty of Veterinary Medicine (approval number 2012–157). All efforts were made to minimize suffering.

Human plasma was obtained from adult subjects who provided a written informed consent. Ethical Committee approval for the scientific use of these blood samples was not applied, according to the Belgian legislation 2013-03-19/03.

### Peptide selection

The currently known quorum sensing peptides are continuously collected into the Quorumpeps database (http://quorumpeps.ugent.be) [26]. To select chemically diverse quorum sensing peptides for BBB-permeability investigations, we optimized the three-dimensional structure of these 231 peptides (status in August 2014) and calculated over 3000 descriptors for each peptide. After removal of the constant descriptors, and correction for molecular weight, a final dataset of 1468 descriptors was retained. Multivariate data-analysis on this resulting 231×1468 data-matrix was performed using Principal Component Analysis (PCA) with SIMCA-P+ 12.0 (Umetrics, Sweden) and different clusters identified [27]. Finally, three chemically diverse quorum sensing peptides were selected to investigate their brain permeability characteristics.

### Peptide handling

The quorum sensing peptides were purchased at GL Biochem (Shangai, China) and the positive control dermorphin at Bachem (Bubendorf, Switzerland). The peptide purity was determined to be ≥ 90%, based on UPLC-PDA analyses [28]. Prior to experimental use, the peptides were dissolved in phosphate buffer (25 mM) at a concentration of 1 μmol/ml.

### Peptide ^125^I radiolabeling and purification

Dermorphin and BIP-2 were labeled using the Iodogen method [29,30]. Briefly, 0.1 μmol of the lyophilized peptide was dissolved in 100 μl of phosphate buffer (pH 7.4, 25 mM). A Iodo-Gen® coated tube was previously rinsed with 1 ml of phosphate buffer. Subsequently, 50 μl of sodium iodide solution (1.1 μmol/ml) and 1 mCi of Na^125^I solution were transferred into this Iodo-Gen® coated tube. The oxidation reaction was allowed to proceed for six minutes at room temperature, after which the iodonium solution was transferred to 50 μl of peptide solution (1 μmol/ml). The iodination reaction of the peptide was allowed to proceed another six minutes at room temperature. Next, the reaction mixture was analyzed by radio-HPLC and the eluting fractions determined for radioactive content (*i*.*e*. peptide concentration). The radio-HPLC apparatus consisted of a LaChrom Elite L-2130 pump with degasser (flow rate is 1 ml/min), a LaChrom Elite L-2300 column oven set at 30°C, a LaChrom Elite L-2400 UV-detector set at 215 nm (all Hitachi, Tokyo, Japan), a Rheodyne 7725i manual injector with 100 μl sample loop (Rheodyne, Rohnert Park, CA, USA), a Berthold LB500 HERM radioactivity detector (Berthold Technologies, Bad Wildbad, Germany) equipped with EZChrom Elite version 3.1.7 software for data acquisition (Scientific Software, Pleasanton, CA, USA) and a fraction collector FC 203 (Gilson International BV, Den Haag, The Netherlands). For separation, a Vydac Everest C_18_ (250 × 4.6 mm, 5 μm particle size) column (Grace, Baltimore, MD, USA) was coupled to the HPLC system. Mixtures of water (0.1% FA m/V) and acetonitrile (0.1% FA m/V) were used to create appropriate gradients for separation of peptides and their iodinated forms. The mono- (and di-) iodinated peptide fractions were then concentrated (if necessary) by nitrogen drying and the appropriate peptide concentrations prepared using Lactated Ringer’s solution containing 1% of BSA. The negative control, *i*.*e*. BSA, was iodinated using the same procedure and the iodinated protein isolated from free iodine using an argent filter.

Peptides phrANTH2 and phrCACET1 were iodinated using the Chloramine-T (CAT) method [31]: 50 μl of peptide solution (1 μmol/ml) was subsequently mixed with 20 μl of 4.5 mg/ml NaI in 100 mM phosphate buffer (phrANTH2) or 0.1% m/V formic acid in water (phrCACET1), 1 mCi of Na^125^I solution and 30 μl of a 4 mg/ml CAT in 100 mM phosphate buffer solution. For phrCACET1, 40 μl of 0.1% m/V formic acid in water was added before the CAT solution as well. The iodination reaction was continued for 120 (phrANTH2) or 40 (phrCACET1) seconds, after which 30 μl of sodium metabisulphite solution (8 mg/ml) was added to neutralize the oxidizing agent. Next, the reaction mixtures were analyzed by radio-HPLC using the described procedures and the eluting fractions determined for radioactivity amount. Again, nitrogen drying was performed on the mono- (and di-) iodinated fractions and the solutions prepared using Lactated Ringer’s solution containing 1% of BSA.

### Multiple time regression analysis

In order to determine whether the peptides could enter the brain, *in vivo* multiple time regression (MTR) analyses were performed. Therefore, ICR-CD-1 mice were anesthetized intraperitoneally using a 40% urethane solution (3 g/kg). Then, the jugular vein and carotid artery were isolated and 200 μl of the radiolabeled peptide solution, diluted to 30 000 cpm/μl using Lactated Ringer’s solution containing 1% of BSA (LR/BSA), was injected into the jugular vein. At specified time points after injection (*i*.*e*. 1, 3, 5, 10, 12.5 and 15 min, with start and end in duplicate), blood was obtained from the carotid artery followed by decapitation of the mouse. The isolated brain was weighed and radioactivity measured in a gamma counter (Wallac Wizard automatic gamma counter, Perkin Elmer, Shelton, CT, USA), as well as from 50 μl serum, which was obtained by centrifuging the collected blood at 10 000 *g* for 15 min at 21°C. To evaluate the tissue distribution of the peptides during the BBB-experiments, seven other tissues, *i*.*e*. spleen, kidneys, lungs, heart, duodenum, muscles and liver, were collected immediately after decapitation of the mice at the last time point of 15 min. After weighing the tissues, the radioactivity was measured in a gamma counter.

The linear modeling of the multiple time regression analysis is based on the Gjedde-Patlak equation [32–34]:

$$\frac{A_{m}(T)}{C_{p}\;(T)}=K_{in}\;\Theta +V_{i}\;\text{where}\;\Theta =\int_{0}^{T}\frac{C_{p}\;(t)\;dt}{C_{p}\;(T)}$$ (1)

and where A_m_(T) is the amount of radioactivity in the brain at time T, C_p_(T) the amount of radioactivity in serum at time T, K_in_ the brain influx rate constant, Θ the “exposure” time and V_i_ the initial brain distribution volume. The exposure time represents the theoretical steady-state serum level of radiolabeled peptide at the serum concentration C_p_(t) and is defined as the integral of the serum radioactivity over time divided by the radioactivity at time T.

During the multiple time regression experiments, peptides are intravenously injected and subsequently cleared by the organs. Therefore, the exposure time is used during the modeling of the brain influx of the peptides to account for the decreasing concentrations. The integral of radioactivity over time is represented by the area under the curve (AUC) [32,35,36].

Finally, the brain/serum ratios (μl/g) were plotted versus the exposure time and the slope of this relationship represents the unidirectional influx rate (K_in_) from blood to brain and the intercept represents the initial brain volume of distribution (V_i_).

If within the experimental time frame, there is a transition from unidirectional to net transfer, then the following expansion of the Gjedde-Patlak plot, a model of biphasic blood-brain transfer as derived from Wong et al [37], was used to fit the uptake:

$$\begin{array}{l} \frac{A_{m}\left(T\right)}{C_{p}\left(T\right)}=K\Theta +\;V_{g}\;\left(1-e^{\left(-\Theta \left(\frac{K_{1}-K}{V_{g}}\right)\right)}\right)+V_{0}\\ \;If\;K=0,\;then\;\frac{A_{m}\left(T\right)}{C_{p}\left(T\right)}=V_{g}\;\left(1-e^{\left(-\Theta \left(\frac{K_{1}}{V_{g}}\right)\right)}\right)+V_{0} \end{array}$$ (2)

Where K_1_ is the unidirectional clearance, K is the net clearance, Θ the exposure time, V_g_ the tissue brain distribution volume and V_0_ the vascular brain distribution volume, experimentally determined by the lowest V_0_ obtained, *i*.*e*. from PhrANTH2, which was somewhat lower than the standard vascular marker iodinated BSA [38].’

For the evaluation of the tissue distribution of the radiolabeled peptides 15 min after IV-injection, the percentage of the injected dose for each isolated tissue was calculated as follows:

$$\%\;injected\;dose=\;\frac{A_{tissue}/w_{tissue}}{A_{injected}/w_{animal}}\;x\;100$$ (3)

where A_tissue_ and A_injected_ are the measured activities of the isolated tissue and the activity of 200 μl of MTR solution, respectively, while w_tissue_ is the weight of the selected tissue and w_animal_ is the mass of the injected mouse. The results are the mean values of the duplicates.

### Capillary depletion

We performed capillary depletion to determine whether the peptides, taken up by the brain, completely crossed the capillary wall into the tissue rather than just being trapped in the endothelium. The method of Triguero *et al*., as modified by Gutierrez *et al*., was used [39,40]. Briefly, ICR-CD-1 mice were first anesthetized intraperitoneally using a 40% urethane solution (3 g/kg). After isolation of the jugular vein, 200 μl of the iodinated peptide solution, diluted to 10 000 cpm/μl using LR/BSA, was injected in the jugular vein. Ten minutes after injection, blood was collected from the abdominal aorta and the brain was perfused manually with 20 ml of Lactated Ringer’s buffer after clamping the aorta and severing the jugular veins. Subsequently, the brain was collected, weighed and the radioactivity measured in the gamma counter. Then, the brain was homogenized with 0.7 ml of ice-cold capillary buffer (10 mM HEPES, 141 mM NaCl, 4 mM KCl, 2.8 mM CaCl_2_, 1 mM MgSO_4_, 1 mM NaH_2_PO_4_ and 10 mM D-glucose adjusted to pH 7.4) in a pyrex glass tube and mixed with 1.7 ml of 26% ice-cold dextran solution in capillary buffer. The resulting solution was weighed and centrifuged in a swinging bucket rotor at 5400 *g* for 30 min at 4°C, after measuring the radioactivity in the gamma counter. Pellet (capillaries) and supernatant (parenchyma and fat tissues) were collected, weighed and measured in a gamma counter. After centrifuging the obtained blood (10 000 *g*, 21°C, 15 min), the radioactivity of 50 μl serum was measured in a gamma counter as well.

Compartmental distribution was calculated as follows:

$$Fraction=\;\frac{CD_{tissue}}{\frac{A_{capillaries}}{A_{serum}}+\frac{A_{parenchym}}{A_{serum}}}\;x\;100$$ (4)

where CD_tissue_ represents the ratio of the activity of the capillaries or parenchyma and the activity of serum for the fraction of radiolabeled peptide in the capillaries and parenchyma, respectively.

### Brain-to-blood transport

We quantified the amount of peptide exported out of the brain as previously described [41]. ICR-CD-1 mice were anesthetized intraperitoneally using a 40% urethane solution (3 g/kg). Then, the skin of the skull was removed and a hole was made into the lateral ventricle using a 22 G needle marked with tape at 2 mm at the following coordinates: 1 mm lateral and 0.34 mm posterior to the bregma. The anesthetized mice received an intracerebroventricular (ICV) injection of 1 μl of the diluted iodinated peptide solution using LR/BSA (25 000 cpm/μl) by pumping the peptide solution at a speed of 360 μl/h for 10 s using a syringe pump (KDS100, KR analytical, Cheshire, UK). At specified time points after ICV-injection (*i*.*e*. 1, 3, 5, 10, 12.5 and 15 min), blood was collected from the abdominal aorta and subsequently the mouse was decapitated. Then, the whole brain was collected, weighed and measured in a gamma counter, as well as from 50 μl of serum, which was obtained by centrifuging the collected blood at 10 000 *g* during 15 min at 21°C. The efflux half-life was calculated from the linear regression of the natural logarithm of the residual radioactivity in brain versus time as follows:

$$t_{1/2}=\;\frac{\text{ln}\left(2\right)}{k_{out}}$$ (5)

where k_out_ is defined as the efflux rate constant calculated as the negative value of the slope of the linear regression, applying first order kinetics.

### *In vitro* human plasma stability

*In vitro* metabolic stability of the quorum sensing peptides was determined in human plasma using previously described procedures [42]. In brief, 100 μl of non-radiolabeled peptide (1 mg/ml) was incubated in 400 μl of Krebs-Henseleit buffer pH 7.4 and 500 μl of plasma at 37°C while shaking. At predetermined time intervals (*i*.*e*. 0, 30 and 120 minutes), 100 μl aliquots were immediately transferred into microtubes containing 100 μl of 1% (V/V) trifluoroacetic acid solution in water. The enzyme reaction was further stopped by heating the solution at 95°C for 5 minutes. Next, the samples were cooled for 30 minutes in ice and subsequently centrifuged to precipitate the denatured proteins; the supernatant was analyzed using UPLC-PDA/MS. The system consisted of a Waters Acquity H-Class Bio-samples FTN (flow rate set at 0.5 ml/min), Waters Acquity H-Class BioQuaternary Solvent Manager, Waters Acquity H-Class column module (set at 30°C), Waters Acquity H-Class Photodiode Array Detector (PDA, quantification at 210 nm) or Waters Xevo TQ-S (Selected Ion Recording, SIR) and equipped with Waters Empower Pro software version 2 or MassLynx version 4.1 (Waters, Zellik, Belgium). Mixtures of water (0.1% FA m/V) and acetonitrile (0.1% FA m/V) were used to create appropriate gradients for separation of peptides and their metabolites. An appropriate placebo solution was similarly prepared. Assuming first-order kinetics, the rate constant k was obtained from

$$\text{ln}\frac{P_{t}}{P_{t0}}=-kt$$ (6)

from which the half-life was determined as

$$t_{1/2}=\frac{\text{ln}\left(2\right)}{\text{k}}$$ (7)

## Results

### Peptide selection

**Fig 1:**
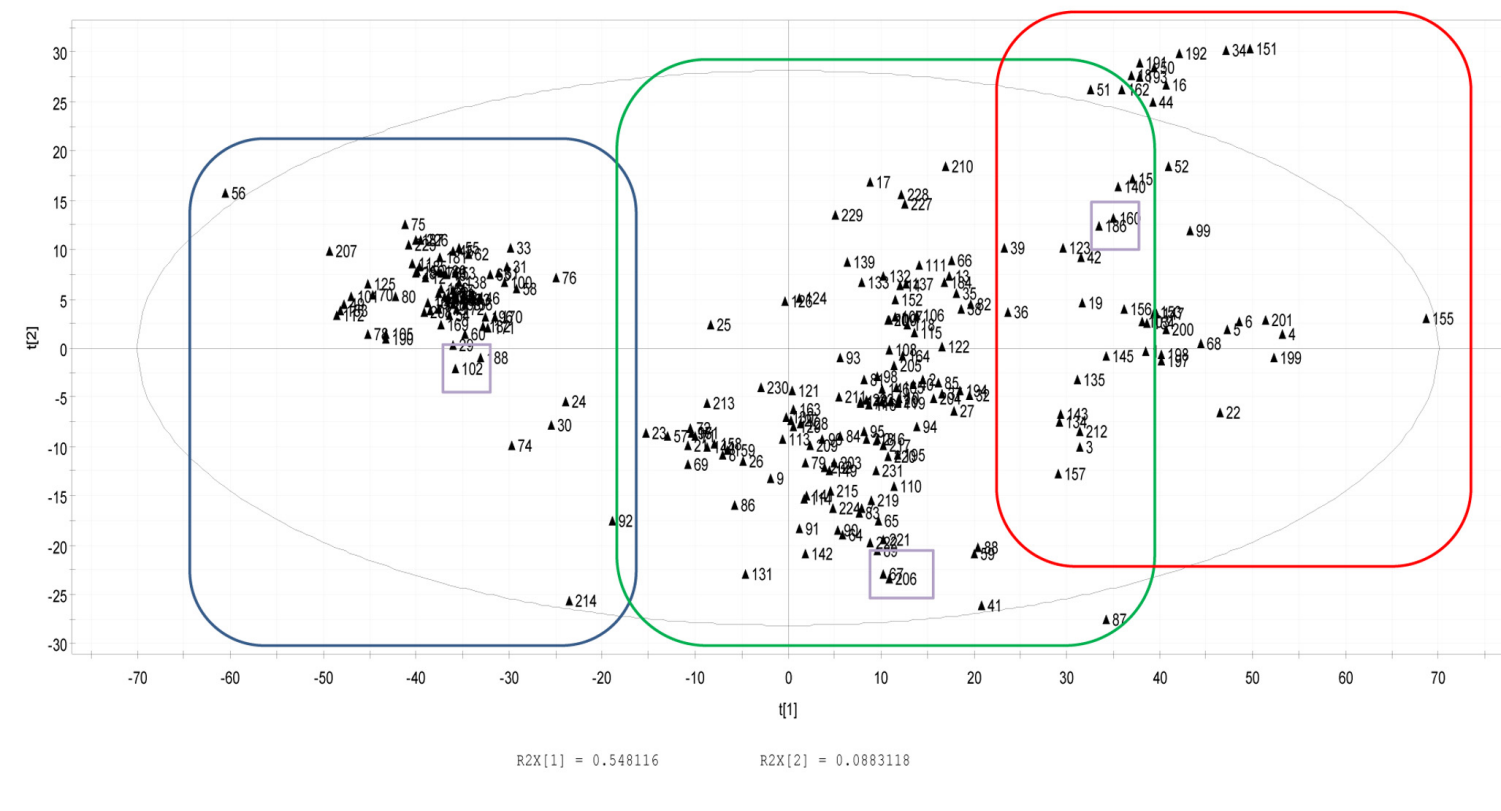
Score plot of the PCA analysis, distinguishing 3 main clusters. The selected peptides are indicated by the rectangles, *i*.*e*. Quorumpeps ID 102 (BIP-2), ID 206 (PhrCACET1) and ID 186 (PhrANTH2).

The clustering results of the quorum sensing peptides are given in Fig 1: three main, chemically diverse clusters can be distinguished [27]. One peptide from each cluster was selected, resulting in three chemically diverse molecules, *i*.*e*. BIP-2 (Quorumpeps ID 102, GLWEDLLYNINRYAHYIT), PhrANTH2 (Quorumpeps ID 186, SKDYN) and PhrCACET1 (Quorumpeps ID 206, SYPGWSW). BIP-2, or bacteriocin-inducing peptide 2, is synthesized by *Streptococcus pneumonia*, a commensal of the human nasopharynx [43,44]. PhrANTH2 is produced by *Bacillus anthracis*, while PhrCACET1 is formed by *Clostridium acetobutylicum* [45].

### Brain influx and tissue distribution

**Fig 2:**
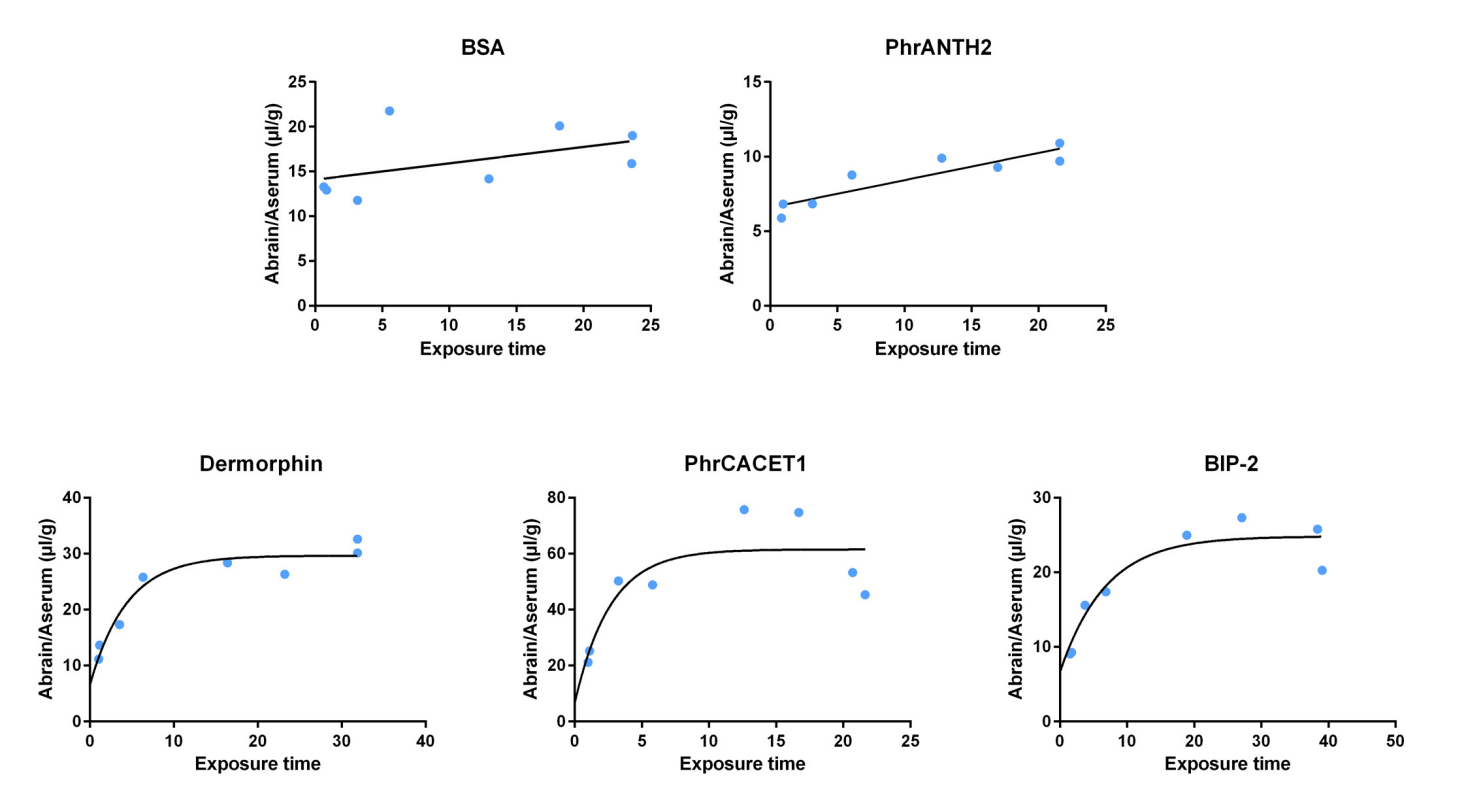
Brain influx of the selected quorum sensing peptides, using the simple linear regression method for BSA and PhrANTH2 and biphasic model for dermorphin, PhrCACET1 and BIP-2. BSA and dermorphin were used as the negative and positive controls, respectively. PhrANTH2 shows no significant brain influx, while the results of PhrCACET1 and BIP-2 indicate BBB permeability, which is the highest for PhrCACET1.

Two of the three investigated quorum sensing peptides showed a statistically significant influx into the mouse brain. It is also clear that dermorphin, PhrCACET1 and BIP-2 showed a biphasic brain influx model [46], characterized by a rapid initial uptake followed by a plateauing equilibrium. In Fig 2, the ratio of the brain and serum radioactivity is plotted versus the exposure time; the quantitative influx parameters of the molecules are summarized in Table 1. The data were fitted using a simple linear regression model (Eq 1) for BSA and PhrANTH2 and a biphasic model (Eq 2) for dermorphin, PhrCACET1 and BIP-2.

**Table 1:** Overview of the multiple time regression results using both the linear as the biphasic model (mean ± 1 standard error).

Peptide (^125^I-)	K_in_ (μL/g × min)	Brain distribution volume, V_0_ (μL/g)	Brain distribution volume, V_g_ (μL/g)	Parenchymal fraction (%)	Capillary fraction (%)	k_out_ (min^-1^)
BSA	0.12 ± 0.08	14.59 ± 1.23	N/A^**b**^	80.6	19.4	0.01 ± 0.03
Dermorphin	5.26 ± 0.93	N/A^**a**^	23.05 ± 1.18	63.0	37.0	-0.11 ± 0.02
PhrCACET1	20.87 ± 7.97	N/A^**a**^	54.90 ± 5.75	84.9	15.1	-0.02 ± 0.04
BIP-2	2.68 ± 0.66	N/A^**a**^	18.22 ± 1.43	76.9	23.1	-0.06 ± 0.02
PhrANTH2	0.18 ± 0.03	6.59 ± 0.47	N/A^**b**^	79.4	20.6	-0.05 ± 0.07

^a^ N/A (V_0_): V_0_ was set to be equal to the V_0_ of PhrANTH2

^b^ N/A (V_g_): not applicable

The positive control peptide, dermorphin, clearly showed an influx with a rate constant of 5.26 μl/(gxmin). It is well established that BSA, the negative control protein, shows a very small, almost negligible brain influx [47], which was confirmed here (K_in_ of 0.12 μl/(g×min)). Both controls thus indicated a good performance of the brain influx experiments and their obtained K_in_ values can be used to benchmark the influx results of the quorum sensing peptides. Peptide PhrANTH2 showed a very small influx into the brain, comparable to BSA: a K_in_ of 0.18 μl/(g×min) was obtained, *i*.*e*. statistically not significantly higher than the K_in_ of BSA. Peptide PhrCACET1 showed a very high initial influx into the brain with a K_in_ of 20.87 μl/(g×min); the high V_g_ value indicates a rapid influx as well. BIP-2 showed a small initial influx into the brain with a K_in_ of 2.68 μl/(g×min), which is statistically significantly higher than the K_in_ of BSA and comparable to dermorphin (not significantly different), but lower than the K_in_ of PhrCACET1.

These initial brain influx results thus correspond well with the overall classification conclusions [48]: PhrCACET1 showed the highest brain influx, followed by BIP-2 and dermorphin.

**Fig 3:**
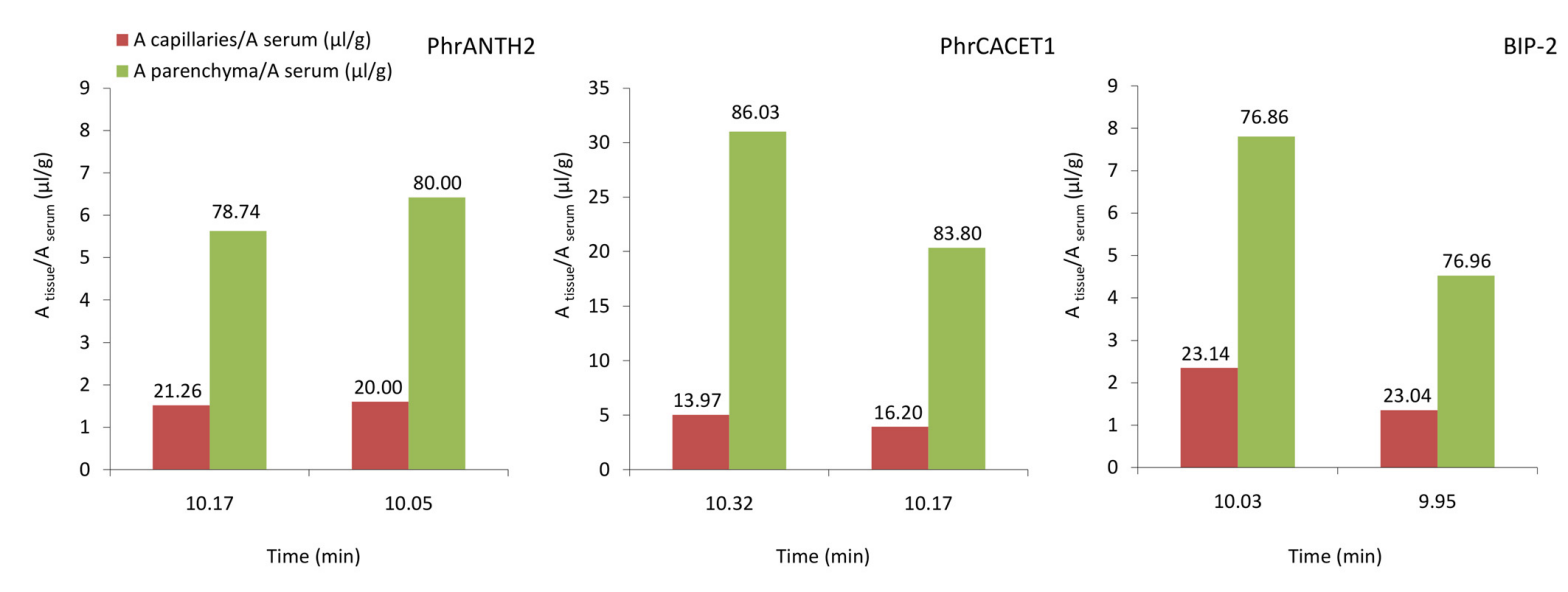
Capillary depletion results of the quorum sensing peptides. The amount of peptide that is effectively transferred into the brain shows a higher brain parenchyma versus a relatively low capillary distribution for the three peptides. The capillary and parenchyma fraction (%) are indicated above the histogram.

The results of the capillary depletion study (Fig 3) at 10 min after injection validate the high brain influx of peptide PhrCACET1: the absolute amount of peptide in the brain (*i*.*e*. absolute y-axis values of Fig 3) is much higher for this peptide compared to the others. The relative amount of peptide that is effectively transferred over the capillaries into the brain showed a higher brain parenchyma (77%–85%) versus a lower capillary retention (15%–23%) for the three peptides (Table 1).

**Fig 4:**
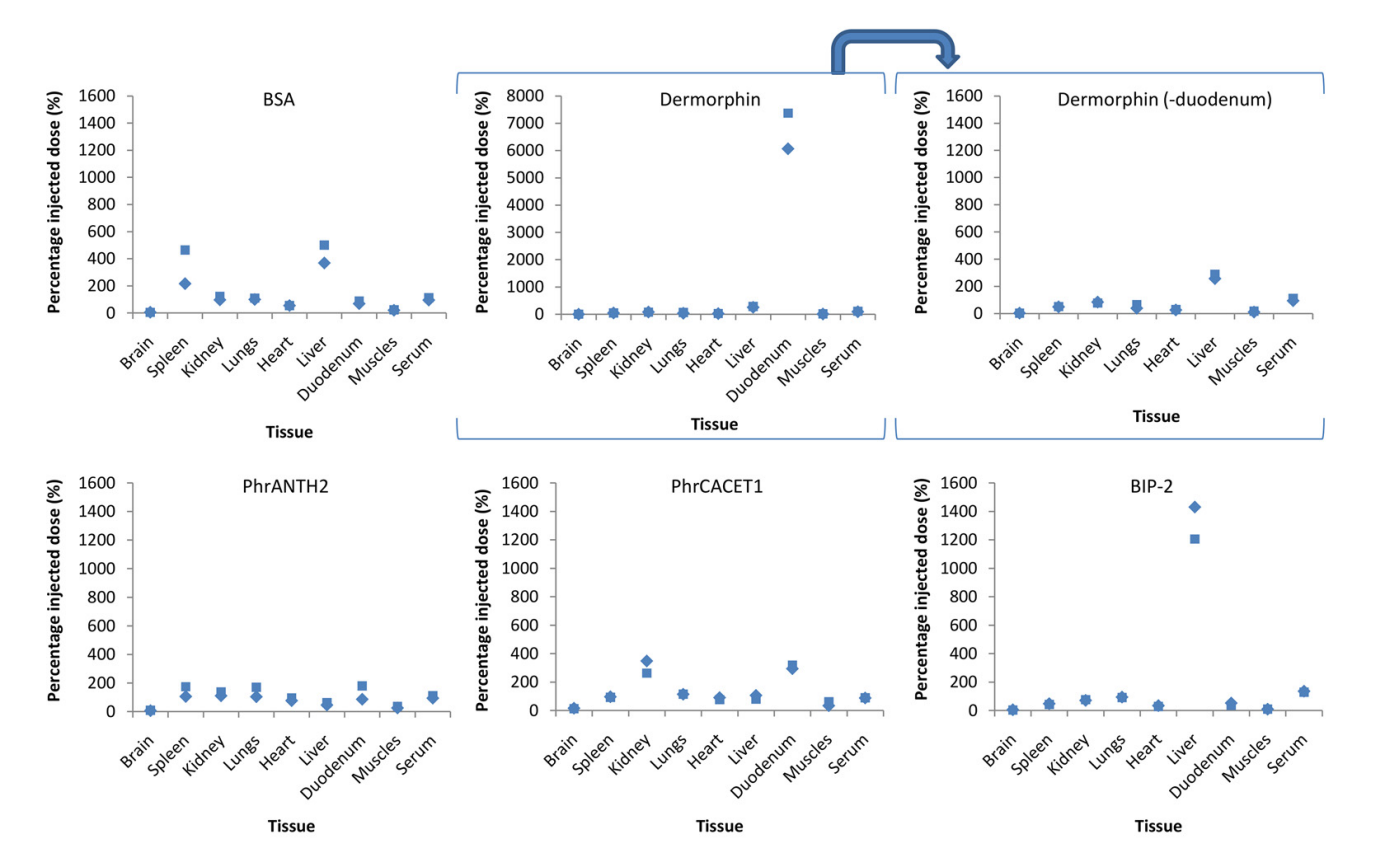
Tissue distribution of the quorum sensing peptides, 15 minutes after IV administration. BSA shows a higher spleen and liver distribution, while dermorphin is massively accumulated in the mice duodenum. PhrANTH2 shows no clear tissue distribution, while PhrCACET1 and BIP-2 are mainly distributed to the kidneys/duodenum and liver, respectively.

In Fig 4, the relative concentrations in the different tissues, obtained from MTR experiments at the last time points (*i*.*e*. at 15 minutes post-injection) are graphically presented. BSA showed a clear liver distribution, while dermorphin was mainly distributed in the duodenum, followed by the liver, 15 min after i.v. injection. The high duodenum accumulation of this control peptide is explained by the presence of peripheral mu opioid receptors for which dermorphin possesses high affinity [49]. PhrCACET1 showed a higher distribution in the kidneys and duodenum compared to the other investigated tissues, while BIP-2 was strongly accumulated in the liver.

### Brain-to-blood transport

The natural logarithm of the measured residual radioactivity in the brain was plotted versus the time, to calculate the efflux constant k_out_. No significant efflux was observed during the experimental time of 15 minutes for the three quorum sensing peptides (k_out_ BIP-2 = -0.06 ± 0.02; k_out_ PhrANTH2 = -0.05 ± 0.07; k_out_ PhrCACET1 = -0.02 ± 0.03).

### *In vitro* human plasma stability

In order to correctly interpret the BBB permeability results, we investigated the stability of these quorum sensing peptides in human plasma. All three peptides were found to be stable during the experimental set-up time (*i*.*e*. maximum 15 minutes) although quite different metabolization kinetics were observed: a half-life value of 56.4, 23.6 and 320.7 minutes was obtained for PhrANTH2, PhrCACET1 and BIP-2, respectively.

## Discussion

The quorum sensing peptide PhrCACET1 (SYPGWSW) very efficiently crossed the BBB, with a measured clearance by the brain that was higher than that of dermorphin (positive control), which generally shows a low but significant clearance at about 0.05% of the plasma flow to the brain [48]. The brain clearance of BIP-2 (GLWEDLLYNINRYAHYIT) was similar to dermorphin, but still higher than the plasma marker BSA. In contrast, PhrANTH2 (SKDYN) shows no significant transport across the BBB in this *in vivo* mouse model. To evaluate these transport results, we compared them with the net clearances of other investigated peptides, obtained using the same technique, for which the data is summarized in the Brainpeps® database [50]. The classification system of BBB influx data, using the BBB_in_ response proposed by Stalmans *et al*. [48], indeed indicated a ‘very high influx’ for the quorum sensing peptide PhrCACET1. A ‘low influx’ classification was obtained for both BIP-2 and dermorphin, while BSA and PhrANTH2 are classified as ‘very low influx’ compounds.

The brain influx of PhrCACET1 and BIP-2 showed a biphasic behavior: after a steep increase of the brain to serum activity ratio, the curves reached a plateau. After this plateau phase, a reduction can not be ruled out. For PhrCACET1, this behavior can be explained by a number of mechanisms, including metabolisation in serum or brain tissue, leading to metabolites with different BBB transport properties as PhrCACET1 has the lowest *in vitro* human plasma stability of the three peptides. Accumulation of the peptide in the kidneys and duodenum can also contribute to this behavior. For BIP-2, the high liver accumulation can influence the brain influx behavior as metabolism of BIP-2 in human plasma was limited during the experimental time frame and thus was not judged to be the primary cause of its observed BBB-kinetic behavior.

The three quorum sensing peptides mainly differ from each other in lipophilicity (principal component 2 in PCA plot) and size/compactness (principal component 1 in PCA plot): lipophilicity is determined by *e*.*g*. the clogP value, the number of hydrogen bonds (nHBonds) and the polarity of the molecule (TPSA), while size is evaluated by *i*.*a*. the WHIM size index and the Wiener W index [27]. As BBB permeability generally increases with lipophilicity, *i*.*e*. PhrANTH2 < BIP-2 < PhrCACET1, our findings are consistent with current physicochemical explanations of BBB transport characteristics of peptides [51]. When we analyze the clustering results of all Quorumpeps peptides (n = 231, Fig 1) [26,27] and select the peptides with a comparable lipophilicity as PhrCACET1 (*i*.*e*. the peptide with the highest BBB permeability characteristics), we mainly find quorum sensing peptides that are synthesized by *Bacillus subtilis*. These peptides thus can, theoretically based on this *in silico* clustering, cross the blood-brain barrier and exert their biological effect in the brain. *Bacillus subtilis*, originally described as a soil organism, is also found in the human gut [52], so these PhrCACET1 lipophilicity-related quorum sensing peptides can be available for the brain once they have reached the blood circulation.

Some quorum sensing peptides, when present in the blood, thus can reach the brain tissue by penetrating the BBB barrier and exert local CNS-effects. BIP-2 is synthesized by the Gram-positive bacterium *Streptococcus pneumoniae*, a commensal of the nasopharynx in the majority of healthy children. Other commensal viridans streptococcal species, such as *Streptococcus mitis*, which are genetically highly related to *Streptococcus pneumoniae*, have commensal nasopharynx and oropharynx properties as well. Next to their commensal behavior, a variety of infectious complications can be assigned to these pathogens, including meningitis, endocarditis, bacteremia and septicemia [44,53]. Based on this bacterial presence, the quorum sensing peptides produced by these genetically related bacteria are expected to be present in the systemic circulation, presented to the brain, followed by BBB-penetration and subsequently biological activity. PhrCACET1 was found in *Clostridium acetobutylicum* [54], but a high genetic homology with other pathogenic Clostridium species, *i*.*e*. *Clostridium botulinum*, *Clostridium perfringens*, *Clostridium difficile* and *Clostridium tetani* [55,56], indicates that comparable quorum sensing peptides can probably also be synthesized in the human body by these bacteria.

Our findings corroborate well with recent clinical findings. The predominant presence of *Clostridium spp*. in the human gut was associated with autism in children [57]. As the quorum sensing peptide PhrCACET1 rapidly crosses the BBB, the neurological effect of this peptide remains to be investigated as well, as these results could potentially explain the mechanisms by which commensal flora trigger autism; the intestinal absorption capability of selected quorum sensing peptides was recently demonstrated [11]. Next to autism, recent studies have indicated that microbiota have dramatic effects on other central nervous dysfunctions as well. For example, a shift in the gut microbial composition was shown in hepatic encephalopathic patients, indicating the possible involvement of the microbiota in the pathophysiology of this disease [58]. Moreover, the human microbiome also plays a role in depression, anxiety and stress [59]. With depression, a decrease in mood disorders was observed with probiotics containing Lactobacilli and Bifidobacteria [60]. The same beneficial effect of these probiotics is observed on anxiety as well; in contrary, infection with *Campylobacter jejuni* elevated anxiety-like behavior [60,19]. An altered gut microbiota composition was also associated with stress: a decreased abundance of *i*.*a*. *Bacteroides* and an increased abundance of *i*.*a*. *Clostridium* was observed after stress exposure [61]. Immune-mediated neuro-psychiatric disorders may be influenced by the microbiota as well, including multiple sclerosis (MS), neuromyelitis optica and Guillain-Barré syndrome [60]. These recent findings, together with our results of BBB-penetration of quorum sensing peptides into the brain, thus excite the research on the influence of these signaling molecules on the development of central nervous system (CNS) disorders.
